# Risk factors for unfavorable clinical outcomes in patients with brain abscess in South Korea

**DOI:** 10.1371/journal.pone.0257541

**Published:** 2021-09-20

**Authors:** Yun Suk Cho, Yu Jin Sohn, Jong Hoon Hyun, Yae Jee Baek, Moo Hyun Kim, Jung Ho Kim, Jin Young Ahn, Su Jin Jeong, Nam Su Ku, Jun Yong Choi, Joon-Sup Yeom, Young Goo Song

**Affiliations:** 1 Department of Internal Medicine, Yonsei University College of Medicine, Seoul, South Korea; 2 AIDS Research Institute, Yonsei University College of Medicine, Seoul, South Korea; Hospital Dr. Rafael A. Calderón Guardia, CCSS, COSTA RICA

## Abstract

**Background:**

Brain abscess can be life-threatening and manifest various neurological findings, although the mortality rate has decreased recently. We investigated the risk factors for unfavorable outcomes of patients with brain abscess.

**Methods:**

A retrospective cohort study examined patients with brain abscess seen from May 2005 to December 2018 in a tertiary care hospital in Seoul, South Korea. We reviewed the medical records for clinical findings, therapeutic modalities, and prognostic factors of brain abscess. Unfavorable clinical outcomes were defined as death, moderate to severe disability with neurological deficits, or vegetative state at 1 year or at the time of discharge from outpatient follow-up.

**Results:**

The study enrolled 135 patients: 65.2% were males; the mean age was 56 years. 35.6% had unfavorable outcomes. In multivariate analysis, higher Sequential Organ Failure Assessment (SOFA) (*p* < 0.001), pre-existing hemiplegia (*p* = 0.049), and higher Charlson comorbidity index (CCI) (*p* = 0.028) were independently associated with unfavorable outcomes.

**Conclusions:**

Higher SOFA, pre-existing hemiplegia and higher Charlson comorbidity index were significant risk factors for unfavorable clinical outcomes in patients with brain abscess.

## Introduction

A brain abscess is a severe purulent infection of the brain, and its worldwide prevalence is 0.4–0.9 persons per 1,000 population [1–3]. Abscesses result from cerebritis, where a capsule forms around the area of inflammation and has a pus-filled center composed of necrotic brain tissue and bacteria. Edema is also increased in the surrounding white matter [3–5].

In the past, brain abscess was invariably fatal. The mortality rate of brain abscess has decreased from 40% to 10% over the past 50 years with the introduction of computed tomography (CT), the development of neurosurgical techniques, and the use of broad-spectrum antibiotics [4, 6–8].

Brain abscess can be life-threatening and have severe neurological sequelae, such as motor weakness, aphasia, and epilepsy [9–11]. Thus, it is important to identify predictors of clinical outcomes of brain abscess. To our knowledge, however, few studies have done this. Therefore, we investigated the risk factors for unfavorable clinical outcomes of patients with brain abscess.

## Methods

### Study population and design

A retrospective cohort study examined patients with brain abscess from May 2005 to December 2018 in a 2,400-bed, tertiary care hospital in Seoul, South Korea. The patients enrolled met the following criteria: (1) aged 18 years or over; (2) symptoms including fever, headache and altered mental status, which are deemed the classical clinical findings of brain abscess [4, 12–15]; (3) brain CT or magnetic resonance imaging findings showing intracranial brain abscess; and (4) evidence of brain abscess seen on aspiration or in microbiological samples. Patients with fungal, mycobacterial, or parasitic infection, and those with other forms of intracranial abscess, such as an epidural abscess or subdural empyema, were excluded [9]. Demographic data, and data on neurological status on admission, clinical presentation, risk factors for an unfavorable outcome, microbiological profiles, neuroimaging findings, treatment modalities, and clinical outcomes were collected from the electronic medical records. Patients were divided into favorable and unfavorable outcome groups, at 1 year or at the time of discharge from out-patient follow-up if earlier. The study was approved by the Institutional Review Board (IRB) of Yonsei University Health System Clinical Trial Center (4-2020-0160). Because the study was retrospective and the data were anonymized, the IRB waived the requirement for consent.

## Definitions

Unfavorable clinical outcomes were defined as death, moderate-to-severe disability with neurological deficits, or vegetative state at 1 year or at the time of discharge from outpatient follow-up. The outcome was assessed using the Glasgow Outcome Scale, a validated global scale used to measure the functional outcome following a brain injury, including brain abscess (Table 1) [16]. The neurological deficits included partial or complete paralysis, partial or complete loss of sensation, seizures, poor cognitive function, or visual defects and dysphasia [17–19]. The Charlson comorbidity index (CCI) [20–22] and Sequential Organ Failure Assessment (SOFA) scores were calculated. The SOFA score comprises six organ system function domains (respiratory, cardiovascular, renal, neurological, hepatic, and hematological) [23–25].

**Table 1:** The Glasgow outcome scales of the patients in this study.

Score	Clinical Meaning	Outcome	No. (%)
**1**	**Death**	**unfavorable**	**14 (10.4)**
**2**	**Neurovegetative state; patient unresponsive and speechless for weeks or months**	**unfavorable**	**4 (3.0)**
**3**	**Severe disability; patients dependent for daily support**	**unfavorable**	**26 (19.2)**
**4**	**Moderate disability; patients independent in daily life**	**unfavorable**	**4 (3.0)**
**5**	**Good recovery; resumption of normal life with minor neurological and psychological deficits**	**favorable**	**87 (64.4)**

### Laboratory tests

Tissue samples were obtained from inflammatory lesions of the frontal, sphenoid, and ethmoid sinuses. The microorganisms were identified using the ATB 32 GN System (bioMérieux, Marcy l’Étoile, France) and MALDI Biotyper (Bruker Daltonics, Bremen, Germany).

### Statistical analysis

Independent *t*-tests were used to analyze continuous variables, and chi-square or Fisher’s exact tests were used for analyzing categorical variables. A multivariate binary logistic regression analysis that included significant predictors in the univariate analysis (*p* < 0.05) was conducted to identify independent predictors of an unfavorable outcome. The Hosmer–Lemeshow test was used to determine goodness of fit for logistic regression models. Variables correlated linearly with other variables were excluded from the multivariate analysis. A *p*-value < 0.05 in the multivariate analysis was considered indicative of statistical significance. The analyses were performed using SPSS (ver. 25.0; IBM, Armonk, NY, USA).

## Results

### Baseline characteristics

The study initially investigated 180 patients, of whom 45 were excluded: 31 with epidural abscesses and 14 with subdural empyema. Finally, 135 patients were eligible for the analysis. Of these, 15 had brain abscesses in multiple lobes. There were 88 males (65.2%) and 47 females (34.8%). Their mean age was 56 years. Common clinical findings were focal neurological deficit (51.9%), headache (45.9%), and fever (29.6%). The most common focal neurological deficits were hemiparesis and dysarthria. The most common comorbidities were diabetes mellitus (17.8%) and solid tumor with or without metastasis (15.6%). Radiologically, 120 (88.9%) had a single lobe abscess and 15 (11.1%) had abscesses in multiple lobes. The underlying source of the brain abscess was identified in 46 patients (34.1%): 17 patients (12.6%) had hematogenous spread, 12 (8.9%) had sinusitis, and 9 (6.7%) had a dental infection. Table 2 shows the patients’ demographic and clinical characteristics.

**Table 2:** Baseline characteristics of the patients with brain abscess.

Variables	Total	Favorable outcome	Unfavorable outcome	*p*-value
	(n = 135)	(n = 87)	(n = 48)	
**Male (%)**	**88(65.2)**	**53(60.9)**	**35(72.9)**	**0.161**
**Age (year, mean±SD)**	**56±14**	**53.9±13.1**	**60.7±14.9**	**0.015**
**Presenting symptoms (%)**				
** headache**	**62(45.9)**	**54(62.1)**	**8(16.7)**	**< 0.001**
** fever (≥ 37.8°C)**	**40(29.6)**	**29(33.3)**	**11(22.9)**	**0.205**
** seizure**	**26(19.3)**	**14(16.1)**	**12(25.0)**	**0.209**
** altered mental status**	**38(28.1)**	**15(17.2)**	**23(47.9)**	**< 0.001**
**Focal neurologic deficit (%)**	**70(51.9)**	**42(48.3)**	**28(58.3)**	**0.263**
**Concomitant meningitis (%)**	**14(10.4)**	**8(9.2)**	**6(12.5)**	**0.547**
**Cerebral hemorrhage (%)**	**8(5.9)**	**3(3.4)**	**5(10.4)**	**0.101**
**Source of infection (%)**				
** sinusitis**	**12(8.9)**	**7(8.0)**	**5(10.4)**	**0.643**
** dental infection**	**9(6.7)**	**5(5.7)**	**4(8.3)**	**0.564**
** eye infection**	**2(1.5)**	**2(2.3)**	**0(0)**	**0.290**
** otitis**	**1(0.7)**	**0(0)**	**1(2.1)**	**0.177**
** head injury**	**5(3.7)**	**2(2.3)**	**3(6.3)**	**0.245**
** hematogenous**	**17(12.6)**	**9(10.3)**	**8(16.7)**	**0.289**
** unknown**	**77(57.0)**	**55(63.2)**	**22(45.8)**	**0.051**
**Site of brain abscess (%)**				
** single lobe**	**120(88.9)**	**76(87.4)**	**44(91.7)**	**0.446**
** multiple lobes**	**15(11.1)**	**11(12.6)**	**4(8.3)**	**0.446**
**Comorbidity (%)**				
** CHF**	**6(4.4)**	**2(2.3)**	**4(8.3)**	**0.103**
** CVA**	**5(3.7)**	**2(2.3)**	**3(6.3)**	**0.245**
** peripheral vascular disease**	**2(1.5)**	**1(1.2)**	**1(2.1)**	**0.673**
** COPD**	**1(0.7)**	**1(1.1)**	**0(0)**	**0.456**
** chronic liver disease**	**13(9.6)**	**7(8.0)**	**6(12.5)**	**0.401**
** chronic renal disease**	**5(3.7)**	**3(3.4)**	**2(4.2)**	**0.832**
** diabetes mellitus**	**24(17.8)**	**12(13.8)**	**12(25.0)**	**0.103**
** non-metastatic solid tumor**	**19(14.1)**	**9(10.3)**	**10(20.8)**	**0.093**
** metastatic solid tumor**	**3(2.2)**	**1(1.1)**	**2(4.2)**	**0.255**
** hematologic malignancy**	**2(1.5)**	**1(1.2)**	**1(2.1)**	**0.673**
** pre-existing hemiplegia**	**11(8.2)**	**1(1.1)**	**10(20.8)**	**< 0.001**
** CCI (mean±SD**	**2.44±2.04**	**1.88±1.7**	**3.48±2.23**	**< 0.001**
**Laboratory data (mean±SD)**				
**WBC (× 10**^**3**^**/μL)**	**10.9±14.6**	**11.4±17.5**	**10±5.4**	**0.721**
**PLT (× 10**^**3**^**/μL)**	**272.9±120.3**	**281.9±120.6**	**255.3±119.2**	**0.100**
** BUN (mg/dL)**	**16.5 ± 14.3**	**15.1 ±12.2**	**19.3 ± 17.2**	**0.087**
** Cr (mg/dL)**	**0.9±0.6**	**0.9±0.7**	**0.9±0.6**	**0.633**
**SOFA (mean±SD)**	**1.8±2.0**	**1.2±1.5**	**3.0±2.2**	**< 0.001**
**Treatment modality (%)**				
** open surgery + antibiotics**	**35(25.9)**	**25(28.7)**	**10(20.8)**	**0.316**
** drainage + antibiotics**	**74(54.8)**	**44(50.6)**	**30(62.5)**	**0.183**
** antibiotics only**	**26(19.3)**	**18(20.7)**	**8(16.7)**	**0.570**

SD, standard deviation; CHF, congestive heart failure; CVA, cerebrovascular attack; COPD, chronic obstructive pulmonary disease; CCI, Charlson comorbidity index; SOFA, Sequential Organ Failure Assessment; WBC, white blood cells; PLT, platelets; BUN, blood urea nitrogen; Cr, creatinine.

Surgery was performed in 109 patients (80.7%), of whom 74 underwent drainage, and 35 received open surgery. All patients were treated with systemic antibiotics and 26 patients were treated only with antibiotics (19.3%) (Table 2). Of these 26 patients, seven did not undergo surgical treatment because they had multiple brain abscesses, in six cases, surgery was not performed because the abscess was smaller than 1 cm, four patients had no surgery due to a critical medical condition, seven patients had abscesses that were difficult to access surgically, and two patients with a high risk of bleeding did not undergo surgery.

### Microbiological characteristics

Causative pathogens were identified in 44 culture-positive brain abscess patients (32.6%). Gram-positive bacteria, particularly *Streptococcus* species, were the most frequently detected pathogens (Table 3).

**Table 3:** Bacteria isolated from patients with culture-positive brain abscesses.

Causative Organism	No. of Cases (%)
	(n = 44)
**Gram-positive**	29 (65.9)
*α-Streptococcus*	8
*Streptococcus anginosus* group	5
Coagulase-negative *Staphylococcus* sp.	5
*Staphylococcus aureus*	5
*Bacillus* sp.	1
*Lactobacillus*	1
*Enterococcus faecium*	1
*Parvimonas mica*	1
*Clostridium bifermentans*	1
*Nocardia* sp.	1
**Gram-negative**	15 (34.1)
*Klebsiella pneumoniae*	8
*Escherichia coli*	2
*Enterobacter cloacae*	2
*Haemophilus aphrophilus*	1
*Pseudomonas aeruginosa*	1
*Fusobacterium nucleatum*	1

### Risk factors for unfavorable outcomes among patients with brain abscess

The outcomes were unfavorable in 48 patients (35.6%) (Table 1): 30 patients had a moderate-to-severe disability, four were in neurovegetative states, and 14 had died by 1 year. In univariate analyses, age (*p* = 0.018), headache (*p* = 0.001), altered mental status (*p* < 0.001), pre-existing hemiplegia (*p* = 0.004), higher SOFA (*p* < 0.001), and higher CCI (*p* < 0.001) were significantly associated with unfavorable outcomes. In the multivariate analysis, higher SOFA [p < 0.001, odds ratio (OR) 1.523, 95% confidence interval (CI) 1.206–1.925], pre-existing hemiplegia (*p* = 0.049, OR 7.652, 95% CI 1.850–68.919), and higher CCI (*p* = 0.028, OR 1.279, 95% CI 1.027–1.594) were independent risk factors for unfavorable clinical outcomes in patients with brain abscess (Table 4).

**Table 4:** Uni- and multivariate analysis of risk factors for unfavorable clinical outcomes in patients with brain abscess.

Variables	Univariate			Multivariate		
	OR	95% CI	p-value	OR	95% CI	p-value
**Age (per 1-year increase)**	**1.034**	**1.006–1.063**	**0.018**	**1.003**	**0.960–1.048**	**0.905**
**Headache**	**0.525**	**0.999–1.002**	**0.001**	**1.000**	**0.998–1.001**	**0.648**
**Altered mental status**	**4.416**	**1.997–9.767**	**<0.001**	**2.384**	**0.734–7.739**	**0.121**
**Pre-existing hemiplegia**	**22.368**	**2.764–181.011**	**0.004**	**7.652**	**1.850–68.919**	**0.049**
**CCI (per 1 increase)**	**1.652**	**1.299–2.102**	**<0.001**	**1.279**	**1.027–1.594**	**0.028**
**SOFA (per 1 increase)**	**1.578**	**1.253–1.987**	**<0.001**	**1.523**	**1.206–1.925**	**<0.001**

OR, odds ratio; CI, confidence interval; CCI, Charlson comorbidity index; SOFA, Sequential Organ Failure Assessment.

## Discussion

We found that independent risk factors for unfavorable clinical outcomes in patients with brain abscess were higher SOFA, pre-existing hemiplegia, and higher CCI. Previous studies have reported some prognostic factors for brain abscess. Zhang *et al*. reported that female gender was associated with a poor outcome, while Larsen *et al*. reported that a low Glasgow coma scale score on admission and comorbidities were related to a poor outcome [2, 26].

In this study, a higher SOFA was an independent risk factor for an unfavorable outcome. The SOFA is used to assess organ dysfunction and failure. Several studies have addressed the relationship between unfavorable outcomes of central nervous system (CNS) infection and the SOFA score [27–29]. An early change in SOFA is a useful early prognostic marker for sepsis and mortality. Nakashima *et al*. found that a high SOFA score was associated with mortality in patients with critical illness, including CNS infection [30, 31]. In a meta-analysis of 25 studies, De Grooth *et al*. revealed a strong association between the SOFA and unfavorable outcomes [25, 32, 33]. Ferreira *et al*. showed that serial evaluation of the SOFA during the first 48 hours could predict the outcome of critically ill patients [34]. The SOFA is a good prognostic measure in patients with severe illness, including brain abscess.

Pre-existing hemiplegia was also an independent risk factor for an unfavorable outcome. Patients with hemiplegia as a result of traumatic accidents, strokes, or brain tumors prior to brain abscess have impaired mobility. McLean *et al*. reported that less mobile patients had low lean mass, reduced physical strength, and an elevated mortality rate [35–37]. If damaged brain tissue is exposed to an infectious agent, the damage is exacerbated, thus increasing the severity of neurological deficits [38–40]. It is not clear why patients with impaired mobility are more susceptible to neurological deficits after brain abscess. However, it is likely that their low stamina and damaged brain tissue increase the probability of sequelae after treatment for brain abscess.

A higher CCI was an independent risk factor for an unfavorable outcome in this study. The CCI is a widely validated simple measure of the prognosis in numerous medical conditions, with significant relationships between the CCI and prognosis in multiple diseases [41–44]. A recent large study reported that the CCI predicted mortality in patients with brain abscess in Denmark from 1982 through 2016 [45].

This study had several limitations inherent to its retrospective design. As with any observational study, unmeasured confounders may have influenced the findings. Also, the single-center design (one tertiary referral hospital) led to the selection of severe cases only. A multicenter study including a large patient sample is needed.

In conclusion, in this study, a higher SOFA, pre-existing hemiplegia, and higher CCI were significantly associated with unfavorable treatment outcomes in patients with brain abscess. Therefore, we need to consider treatment carefully in patients with brain abscess who have these predictors.
